# A Very Rare Disease of Patent Urachus Cyst With Vesico-Umbilical Urinary Fistula in Adults: A Case Report and Short Review

**DOI:** 10.7759/cureus.41503

**Published:** 2023-07-07

**Authors:** Viswarupachari Tanguturi Yella, Sree Sudha Tanguturi Yella, Krishna Sasanka Kota, Sri Hari Tanguturi Yella, Pugazhenthan Thangaraju

**Affiliations:** 1 General Surgery, Santhiram Medical College and General Hospital, Nandyal, IND; 2 Pharmacology, All India Institute of Medical Sciences, Deoghar, IND; 3 Ear, Nose and Throat (ENT) and Head and Neck Surgery (HNS), All India Institute of Medical Sciences (AIIMS) Deoghar, Deoghar, IND; 4 Family Medicine and Critical Care Medicine, Omni Hospital, Hyderabad, IND; 5 Pharmacology and Therapeutics, All India Institute of Medical Sciences (AIIMS) Raipur, Raipur, IND

**Keywords:** umbilical fistulogram, urachal anomalies, vesiculo umbilical urinary fistula, urachal cyst, patent urachus

## Abstract

Rare developmental anomalies known as urachal remnants are brought on by flaws in the foetal developmental process. However, depending on the location and degree of incomplete obliteration, the urachus can undergo a variety of urachal anomalies. An umbilical fistulogram and a voiding cystourethrogram both supported the existence of the adult urachal cyst in this case. To treat the sepsis, we provided the patient with antibiotics first, then a surgical procedure. The entire vesico-umbilical tract with the urachal cyst was removed using the open approach. The excised specimen's histology revealed a foreign body giant cell reaction without any indication of malignancy. The presentation and diagnosis of vesico-umbilical urinary fistula (VUUF) in adults can occasionally be difficult. They happen very rarely. So we began putting forward this case for the same reason.

## Introduction

Urachal anomalies are uncommon, but cases have been frequently reported in children and extremely infrequently in adults [1]. During normal development, the proximal allantois extends into the urogenital sinus, and the remaining allantois is encircled by the umbilical cord, from which it emerges to form the urachus, a fibrous connection between the umbilicus and the apex of the bladder [1]. By the 12th week of development, this fibrous tube-like structure should be filled and sealed off to form the median umbilical ligament. However, depending on the location and degree of incomplete obliteration, the urachus can undergo a variety of urachal anomalies. Patent urachus is a rare condition that manifests as a urinary fistula from the upper border of the urinary bladder to the umbilicus. Distal obstruction of the urinary tract, which is a result of enlarged prostate tissue or urethral stricture, is the cause of urinary leakage from the umbilicus [2]. In this report, we outline the findings of an adult patient who underwent surgery to treat a patent urachus after being diagnosed with the condition through diagnostic tests.

## Case presentation

**Figure 1:**
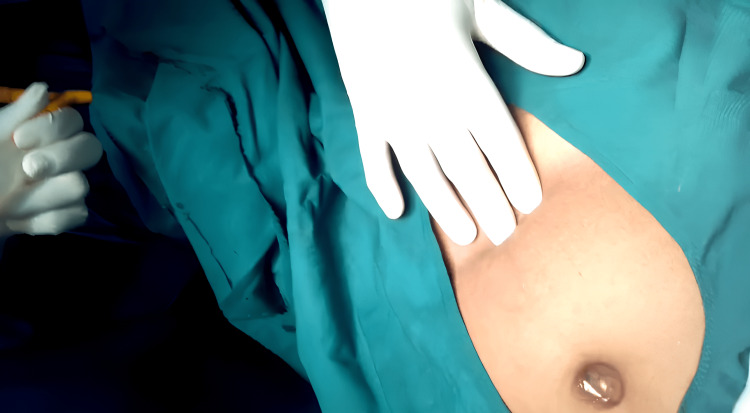
On examination there is opening at the umbilicus Image showing collection of urine at the umbilical site

**Figure 2:**
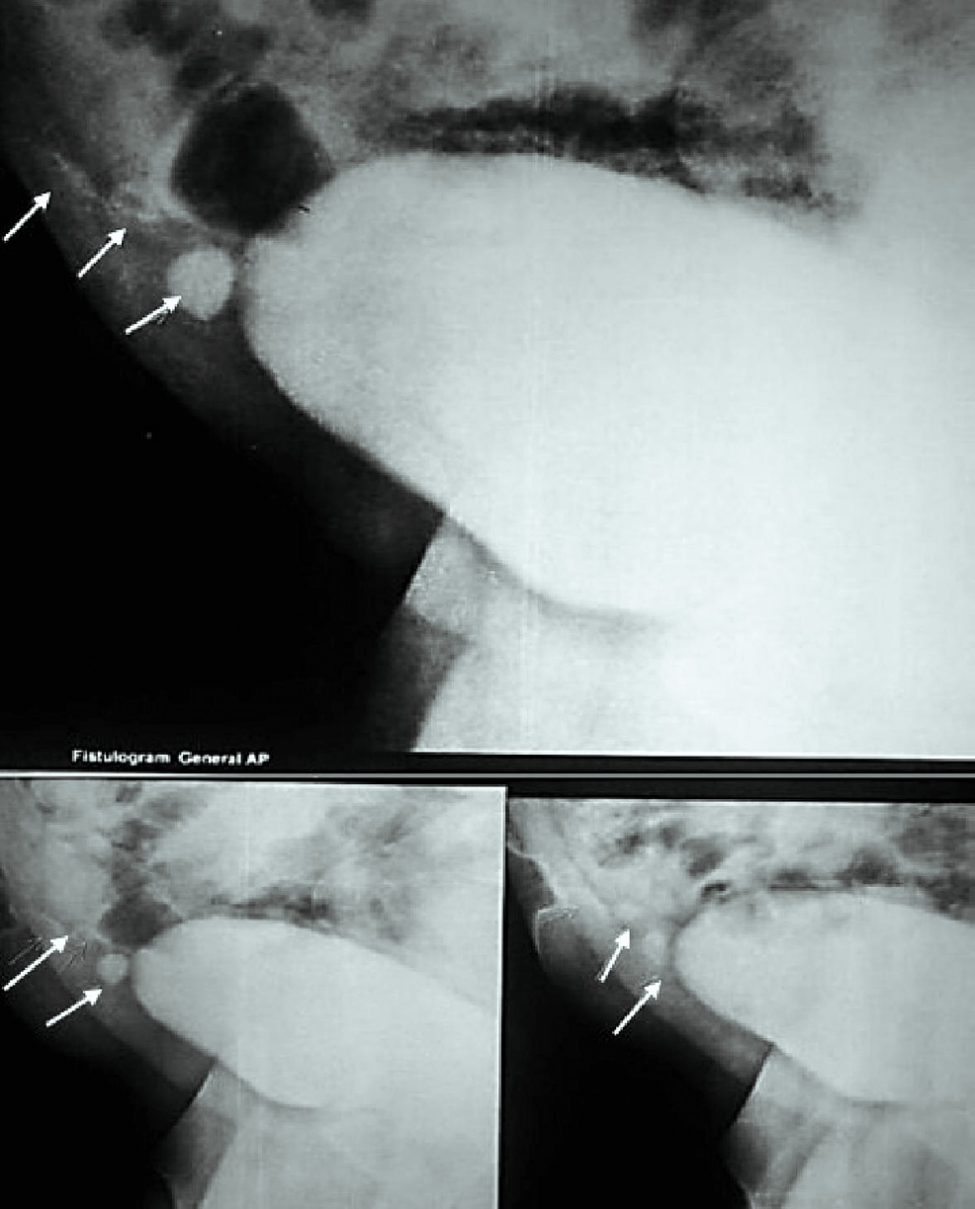
In voiding cystourethrogram, showing a vesicoumbilical fistula with urachal cyst Film showing fistulogram in general AP view

A 48-year-old male patient came in with the main complaint that urine production from the umbilicus had been like a jet for the past three months, with no difficulty emptying the bladder through the urethra. Three days ago, symptoms began, including a fever, colicky-like pain in the hypogastrium, and a thick, yellowish-white discharge with pellets through the umbilicus. These symptoms subsided with symptomatic treatment; there was no prior history of vomiting, nausea, or changes in bowel habits. No abnormalities were found after a systemic review. He was hemodynamically stable upon examination. Upon abdominal examination, it was discovered that the patient had urine dribbling from the umbilicus (Figure 1), mild suprapubic tenderness, mild ascites, and mild hepatomegaly. The prostate and spleen have not shown any abnormalities. A primordial umbilicus disease, a patent urachus, or a urachal cyst were among the working differential diagnoses. A haematological examination is normal. Ultrasound, CT, and MRI were performed to determine the size of the cyst and its relationship with the peripheral tissues. There is an opening at the umbilicus in an umbilical fistulogram (Figure 2). We discovered a vesicoumbilical fistula along with a urachal cyst during a voiding cystourethrogram. Finally identified as having a patent urachus, vesico-umbilical urinary fistula (VUUF), and urachal cyst.

**Figure 3:**
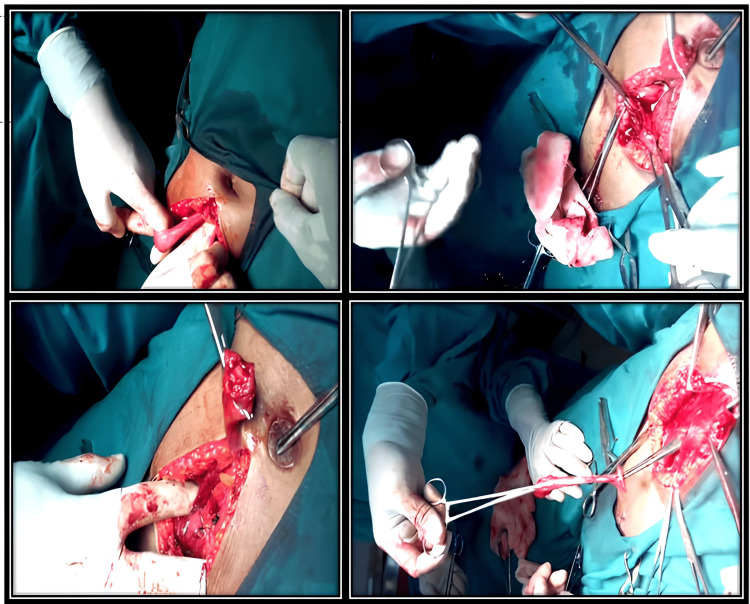
Surgical steps The entire urachus tract including umbilicus was excised along with urachal cyst

**Figure 4:**
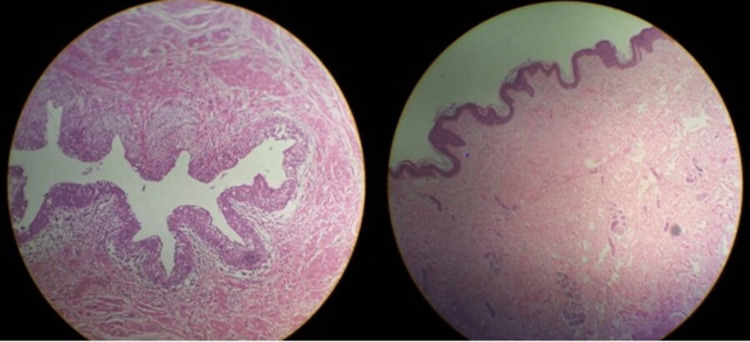
Histopathology showing, the tract was lined by transitional epithelium, which is similar to the bladder epithelium and umbilical section showed keratinized stratified squamous epithelium with a foreign body giant cell reaction No signs of malignancy on histopathology report

A one-week course of antibiotics was administered to the patient, and open surgery was scheduled upon completion of this time. Before surgery, the bladder was saline-distended and clamped using Foley's indwelling catheter. Under spinal anaesthesia, a sub-umbilical midline incision was made through the skin and subcutaneous fascia, keeping the peritoneum unsevered. By blunt dissection of the apex of the bladder, the attachment of the urachus was identified and separated extraperitoneally. Through a racket incision around the umbilicus, the entire tract was separated and dissected. The urachus was divided at the apex of the bladder, and the cut end of the urachus was clamped. The opening of the bladder was closed extramucosally with a chromic 1-0 continuous suture in two layers. The entire urachus tract, including the umbilicus, was excised along with the urachal cyst (Figure 3). Haemostasis was secured, and wound closure was done in layers. A histopathological examination showed that the tract was lined with transitional epithelium, which is similar to the bladder epithelium, and the umbilical section showed keratinized stratified squamous epithelium with a foreign body giant cell reaction. There were no signs of malignancy. The specimen showed chronic inflammation with no evidence of chronic diseases like tuberculosis/Crohn’s or malignancy (Figure 4). Foley's catheter was secured for five days. Sutures were removed on the eighth postoperative day. Bladder wash was given on the day of surgery and the second and third days postoperatively. Recovery was uneventful. The patient has been free from symptoms for the last year and until now.

## Discussion

Umbilical remnants develop when the foetal bladder and allantois are partially or completely obliterated. Complete communication between the bladder and the umbilicus is indicated by a patent urachus. The remnants found in neonates younger than six months usually resolve spontaneously without the need for surgery [3]. Urachal anomalies are more commonly found in males and are rarely observed in adulthood [4]. In adults, management is required because of the greater risk of infection spreading to the peritoneal cavity. Other complications include uracho-colonic fistula, stone formation, and chances of neoplastic transformation [4-6]. The risk of urachal malignancies is very high, with a poor prognosis. The treatment of the urachal cyst involves primary excision of the cyst. The traditional treatment of an infected urachal cyst is composed of a two-stage approach, i.e., an incision and drainage of the infected cyst followed by secondary excision. Single-stage approach, i.e., complete excision of the urachal cyst, urachal duct, and infected umbilicus with the closure of the bladder [7]. McCollum et al. compared the single-stage approach with the two-stage approach and concluded that single-stage excision patients experienced complications, but all the two-stage approach patients were free of complications [8].

In our case, urine flowed out as a jet from the umbilicus along with the normal micturition from the urethra. This is not secondary to a distal obstruction. This was a very rare phenomenon. There was no suspicion of malignancy with a negative history of hematuria. During primary diagnosis, ultrasound, CT, and MRI were performed to determine the size of the cyst and its relationship with the peripheral tissues. In the presence of umbilical discharge, an umbilical fistulogram was performed to confirm the existence of the fistula, and a voiding cystourethrogram was performed for the reflux. With the above symptoms, signs, and other investigation reports, the case was primarily diagnosed as a patent urachus cyst with a vesico-umbilical urinary fistula. The treatment of choice for the above diagnosis is a complete primary excision in a single-stage approach. The open surgical approach is advantageous over the laparoscopic approach, in which there is a chance of incomplete excision and exploration, local hematoma, entry into the peritoneal cavity, and complications of peritonitis. The surgical approach is necessary for the confirmation of the diagnosis and the need for excision [9]. Histopathology revealed that the tract was lined with transitional epithelium, which is similar to the bladder epithelium, and the umbilical section showed keratinized stratified squamous epithelium with a foreign body giant cell reaction with no signs of malignancy. Finally, we can conclude that "vesico-umbilical urinary fistula" with a urachal cyst communicating with the umbilicus is a very rare phenomenon in adulthood. The patient's history, physical examination, and other investigations are crucial for the correct diagnosis and to define the surrounding anatomical relationship. Complete surgical excision by the open method is the treatment of choice. We recommend early diagnosis with appropriate broad-spectrum antibiotic therapy followed by a single-stage surgical approach as the ideal choice of treatment.

## Conclusions

A patent urachus is an extremely rare urachal remnant. It usually presents in the neonatal period with urinary discharge from the umbilicus. In our case, the extraperitoneal approach was preferred instead of laparotomy to avoid the risk of recurrences in the laparoscopic approach and attendant laparoscopy complications. Umbilical cord cysts and large umbilical cords should be evaluated carefully to distinguish between coexisting conditions. We present a case in which an adult with a patent urachus fistula was successfully treated.
